# Progress in Surgery

**Published:** 1895-06-01

**Authors:** 


### Progress in Surgery.

### SURGERY OF THE LIVER AND GALL BLADDER.

(*Continued from page* 134.)

**Cholelithiasis.**—The indications for surgical intervention in these cases are, says Gersuny,[6] to be measured by the danger resulting from frequent attacks of colic and the prolonged existence of jaundice. Operation may also be necessitated by rupture of the gall bladder, impermeability of the intestine, or incipient septicæmia. If the attacks are very frequent, there is danger of chronic irritation of the gall bladder, which may result in the development of carcinoma. Galliard[7] divides operable cases of cholelithiasis into two classes: the feverish and the apyretic. In *feverish patients*, two conditions may present themselves: (1) A tumour exists, with or without icterus. In this case prompt intervention is a necessity, unless there are obvious contra-indications, such as generalised acute peritonitis, pylephlebitis, the various manifestations of septicæmia, endocarditis, pericarditis, broncho-pneumonia, &c. The best method of operating consists in opening the gall bladder and inserting a drain. Cholecystectomy should only be practised when there is no doubt of the permeability of the ductus choledochus. (2) Infective icterus exists, without tumour. In this infective angio-cholitis surgery is powerless, and there is no justification for operation unless the obstruction of the ductus choledochus is absolutely certain. In *apyretic subjects*, there is no urgency for operation, which is indicated only in case the pain is unbearable, or if the patient is becoming exhausted. When the operation (cholecystotomy, &c.) has been done, it is necessary to determine the permeability or otherwise of the canal. If the ductus choledochus is impervious and dilated, choledocho-duodenostomy may be practised; but this is a difficult operation. Cholecystostomy or cholecystenterostomy are much easier, and the author prefers the former, as it allows calculi which have been overlooked to escape. The contra-indications to operation are pregnancy, extreme hypertrophy of the liver or spleen, hypothermia, pointing to marked deterioration of the liver, and associated with malignant jaundice, tubercular or cardiac cachexia, &c. When ascites exists in connection with retention of bile, the diagnosis must be cancer of the head of the pancreas.

Kehr[8] reports eighty-one operations on fifty-three cases. Of thirty-six patients upon whom he performed cholecystotomy in one or two stages, there were no deaths. In all of the cases a cure has resulted, and in only one instance has a fistula remained. He concludes that: (1) Operation may be necessary, although there be no icterus, swelling of the liver, or tumour of the gall bladder; the pains, also, need not be typical of gall stone colic. 2. In long-standing stomach troubles, such as dilatation, and especially in so-called stomach cramps, one must not lose sight of the possibility of gall stones and of the existence of hernia in the linea alba. 3. Riedel's tongue-shaped appendix is present in many cases of hydrops of the gall bladder; it is not seldom mistaken for wandering kidney on the right side. 4. For removing stones from the gall bladder, cholecystotomy; if the proper technique is employed fistula will not remain. 5. The total extirpation of the gall bladder is to be considered only on account of disease of its walls (carcinoma, ulcerations, &c.), and not on account of the stones that may be contained in it. Dujardin-Beaumetz[9] thinks that cholecystenterostomy presents the serious inconvenience that the bile ducts may be infected by micro-organisms from the intestines. Such infection is manifested by paroxysms of fever, which are the counterpart of those of intermittent fever. Sometimes the attacks occur one by one every four to ten days; sometimes on the contrary they take place during three or four days at a time, recurring at almost exactly the same hour each day. They last from four to six hours. They are preceded by general lassitude, are sometimes accompanied by vomiting and gastric pain, and end with a condition of extreme weakness followed by a characteristic staining (melanoderma) of the tissues. They differ from attacks of intermittent fever in the irregularity of their appearance and their resistance to treatment by quinine. The prognosis is more favourable when the fever does not set in till late—six weeks or two months after the operation. It is only when the gall bladder is transformed into a fistulous canal that there is any hope of seeing the hepatic infection subside, but this does not take place until months, and even years, have elapsed. In spite of its drawbacks, the author believes that cholecystenterostomy must be preferred to cholecystostomy when there is not a reasonable expectation of re-establishing the functions of the ductus choledochus, cholecystostomy being suited only to cases of retention of the bile, due to the existence of an obstacle which may be supposed not to be permanent. In order to get over the difficulty of extracting calculi from the common duct, Anderson[10] has had some forceps constructed with a lock similar to midwifery forceps, so that the two blades may be introduced separately along the narrow ducts. Moffat[11] reports a successful case of cholecystenterostomy by means of Murphy's button.

**Cystico-lithectomy.**—After seventy-seven operations upon the gall bladder, in twenty-six of which he found impacted stones in the ductus cysticus, Kehr[12] proposes incision of the cystic duct in all cases of impaction. Formerly he performed the ordinary cystotomy, leaving a biliary fistula, and after a few months performed a second laparotomy, incised the duct, removed the stone, passed a drainage tube through the duct—now patulous—and sewed the incision. This plan he found open to the objection necessarily raised against a second laparotomy, and he now adopts the following plan: Through a four-inch incision in the right rectus muscle he inserts his right hand, breaking gently all adhesions until the cystic duct is reached. He then searches for and finds the stone, and then endeavours to force it back into the gall bladder, pursuing the method of Lauenstein. Standing on the patient's right side, the operator turns his back to the patient's face, then bending forward, he inserts the left hand into the abdominal opening and endeavours to force the stone from its impacted position. This failing, the gall bladder is aspirated, and if he finds the stone very large or strongly embedded, he proceeds at once to the incision of the ductus cysticus over the point of impaction. The bile and stone are at once removed, drainage inserted, and the incision sewed together with a fine silk and a curved needle.

**Cholecystectomy** has been performed by Richardson[13] in a case of impacted calculus in the cystic duct which it was impossible to remove by other means; and by Briddon[14] for an impacted calculus in the common bile duct.

[6] Med. Week, Nov. 23, 1894. [7] Med. Week, Sept. 14, 1894. [8] Am. J. Med. Sc., Nov., 1894, and Deut. Zeit. f. Chir., 1894, Bd. 38. [9] Med. Week, Aug. 10, 1894. [10] Lancet, Nov. 17, 1894. [11] Med. Rec., Sept. 15, 1894, p. 339. [12] Therapeut. Gaz., Oct. 15, 1894, and Archiv. für Klin. Chir., 1894, vol. 18, No. 3. [13] New York Med. Jour., Nov. 3, 1894. [14] Annals of Surgery, Sept., 1894.

